# Donors’ Participation in Iran’s Health System: Challenges and Solutions

**DOI:** 10.34172/ijhpm.2021.177

**Published:** 2021-12-28

**Authors:** Ali Mohammad Mosadeghrad, Maryam Tajvar, Fatemeh Ehteshami

**Affiliations:** Department of Health Management and Economics, School of Public Health, Tehran University of Medical Sciences, Tehran, Iran.

**Keywords:** Donors, Challenges, Health System, Strategies, Qualitative Study, Iran

## Abstract

**Background:** Philanthropic activities play an important role in health systems. Donors contribute to financing, generating resources, and providing healthcare services in Iranian health system. However, they face many challenges. This study aimed to identify barriers to donors’ participation in the Iranian health system and to provide solutions.

**Methods:** This qualitative study was performed using semi-structured interviews with 38 donors and 26 policy-makers and managers in the social affairs department of health ministry and medical universities in 2018. In addition, document analysis was performed and the relevant data were extracted. Thematic analysis was used for data analysis. All ethical considerations were followed in this research.

**Results:** Insufficient structures, poor communications, low trust, ineffective working processes, bureaucracy, insufficient senior managers’ support, weak legal support and poor monitoring were the most important challenges for donors’ participation in the Iranian health system. Effective donor participation in the health system requires the creation of an appropriate system including the right structures, processes, culture, and management. The necessary changes must be planned, led and monitored to promote donors’ participation in healthcare. A conceptual model was developed to strengthen donors’ participation in the health system.

**Conclusion:** Iranian donors face structural, procedural, cultural, and managerial challenges when financing the health system, generating resources, and providing health services. Policy-makers and managers should tackle these challenges and adopt strategies to reinforce donors’ participation in the health system. Planning, organizing, leading, monitoring, evaluation, transparency, accountability, and a commitment to meet donors’ needs are necessary for successful philanthropy initiatives in the health sector.

## Background

**Key Messages**

** Implications for policy makers**

- Private philanthropy is essential to generate more resources for the health sector. Health policy-makers can take advantage of donors’ participation for financing, generating resources, and providing healthcare services.
- Insufficient structures, poor communications, low trust, ineffective working processes, bureaucracy, insufficient senior managers’ support, weak legal support and poor monitoring and evaluation limit donors’ participation in the Iranian health sector.
- A conceptual model was developed to strengthen donors’ participation in the health system. Health policy-makers and managers can use this model and the proposed strategies to strengthen donors’ participation in the health system.
- Health system priorities should be communicated to donors. The necessary changes to promote donors’ participation in healthcare must be planned and led. The performance of health donors should be evaluated and further actions should be taken if necessary.

** Implications for the public**

Philanthropy is necessary to generate more resources for the health sector. The health sector has always had the support of charities due to its humanitarian nature. Health philanthropists (donors) participate voluntarily in financing the health system, generating resources and providing healthcare services. They play an important role in strengthening the health system and achieving universal health coverage. The Iranian people have always been involved in charity. Addressing donors’ challenges in financing and providing health services will lead to greater public participation in the health sector. Moreover, researchers can use the proposed model to measure the challenges of donors in the health system.

The Islamic Republic of Iran is a middle-income country with a population of about 84 million and per capita gross domestic product of (purchasing power parity) $14 535.^2^ Iran’s health system is hybrid and the public, private and non-profit sectors are involved in financing, purchasing and providing health services. The Ministry of Health and Medical Education is responsible for policy making, financing, planning, and controlling the health sector at the national level. Medical universities are responsible for providing both medical education and healthcare services at the provincial level. The district health network provides primary healthcare services free of charge, and the hospital network delivers secondary and tertiary services. The private and non-profit sectors are more involved in financing and providing specialized health services.^3^

Iran’s health system faces challenges in the areas of equity, efficiency, and quality.^4^ Demand for health services increased due to population growth and an increase in chronic diseases. Furthermore, the use of modern medical technologies and procedures has increased the costs of the Iranian health system. The health system is financed through government general revenues, health insurance premiums, and individuals’ out-of-pocket payments. The total health expenditure of the Iranian people was US$39.6 billion in 2018, which was 0.5% of the world total health expenditure. The per capita health expenditure of the Iranian people was US$484 in 2018, which was 1.2 times the average per capita health expenditure of the world. About 45.9% and 54.1% of Iran’s health expenditures were financed by the public and private sectors, respectively. About 41.7% of this amount has been provided through direct out-of-pocket payment.^2^ The health financing system is regressive, fragmented, inefficient, and inequitable. As a result, the government is facing challenges in financing and providing healthcare services. Private philanthropy may fill this gap and support the health system.

Philanthropy is necessary to generate more resources for the health sector. Eikenberry defines philanthropy as “individual contributions for public good, and to uplift the quality of life.”^5^ Philanthropy is an action to reduce suffering and improve the quality of life and well-being of humanity. The health sector has always had the support of charities due to its humanitarian nature. Health philanthropists (donors), individually or in groups, participate voluntarily in financing the health system, generating resources and providing healthcare services.^6^ Health donors are people who participate in meeting the health needs of the society with good intentions as their social responsibility. They play an important role in strengthening the Iranian health system.

There is a pressing need for sufficient and reliable funding to support health systems. Donor funding has always been a source of health system financing, especially in low- and middle-income countries. Both domestic and international (governments, bilateral and multilateral aid agencies) donors contribute to funding health systems. Individuals and charities around the world contributed about $24.3 billion to the health sector in 2017. Donor funding accounted for about 30% of total health spend­ing in low-income countries. These charitable donations were mostly spent on combating infectious diseases, family planning programs, maternal and child health, strengthening countries’ health systems, and global health security. Most of these charities came from the United States ($10 billion), the United Kingdom ($2.4 billion), Germany ($1.2 billion), France ($971 million), and Japan ($871 million). Most health charities went to Afghanistan, Bangladesh, Ethiopia, Haiti, Kenya, Namibia, Ghana, Rwanda, India, Nigeria, Tanzania, Uganda, Zambia, Pakistan, Sierra Leone and Zimbabwe.^7^

The Iranian people have always been involved in charity. Iran was ranked 29th in the world according to the World Giving Index in 2017. Iran was ranked 10th in the world with a population of 32 million people donating money.^8^ There were about 890 active health charities in Iran in 2018.^9^ Donors are involved in financing, resource generation, and health services delivery in Iran. Donors individually or in groups collect and mobilize the financial resources needed by the health sector and spend on constructing and developing healthcare facilities, purchasing equipment and supplies, paying the salaries of healthcare workers and paying poor patients’ bills.^10^ They are also involved in the delivery of healthcare services.^6^ There are 38 charity hospitals with 4370 beds (3.4% of total hospital beds) in Iran.^11^

 Lack of financial resources puts a lot of pressure on people, health insurance companies and the government especially during times of economic recession. Therefore, the use of charitable resources is essential to strengthen Iran’s health system. On the other hand, donations from health donors are declining due to the recession. As a result, there is a competition between different sectors of the country to attract charitable donations. Therefore, it is essential to be aware of the challenges that donors face in contributing to Iran’s health system. Hence, the purpose of this study is to identify these challenges and solutions to address them. The results of this study provide useful information to health policy makers and managers to strengthen the participation of donors in financing, resource generation and health services delivery.

## Methods

This qualitative and inductive study was performed using interpretive phenomenology method. Phenomenological research is the science of studying, describing, and accurately interpreting life phenomena. In this type of research, people’s experience, perception and feelings about a subject phenomenon are studied.^12^ Interpretive phenomenology is suitable for identifying, examining, describing, explaining and interpreting phenomena, facts, events, processes, activities and concepts of which we have little knowledge.

 Although domestic and international donors are involved in financing the health systems of countries, Iran has less access to international aids due to the political sanctions. Therefore, the present study was limited only to domestic donors. The Iranian donors are involved in financing, generating resources and providing health services in Iran. However, they face some challenges in contributing to the health system. Therefore, in this study, interpretive phenomenology was used to identify, describe, explain and interpret these challenges and solutions to address them.

 Research data was collected through semi-structured interviews with health donors and health policy-makers and managers dealing with charities in Iran’s health sector. In addition, document analysis was performed using 16 related laws, regulations, policies, plans, etc to find rich information about the donors’ challenges and solutions to overcome the challenges.

 Purposeful and snowballing methods were used to identify research participants. The selected individuals had the most knowledge about donors’ participation in the health sector. The use of pluralistic evaluation in this study provides a comprehensive and in-depth knowledge of the barriers to donors’ participation in Iran’s health system and their solutions, which is not possible by conducting interviews with only one group of stakeholders. Interview questions were developed according to the objectives of the research and a review of previous studies. Interviews continued until data saturation (64 interviews). Interviews were conducted by voice recording and note-taking. The average interview time was 14 minutes (minimum 9 minutes and maximum 18).

Thirty-eight health donors and 26 health policy-makers, managers and employees participated in this study. Donors were limited to persons who either directly donate to hospitals and healthcare organizations or indirectly help the health system by contributing to health charitable foundations and non-governmental organizations (NGOs). Table 1 shows the demographic characteristics of the interviewees. Most interviewees were male (68.8%), married (85.9%), over 55 years old (45.3%), had Master’s university degree (23.5%), and had 10-20 years of philanthropic experience. Of the 38 interviewed donors, 13 were active in health service delivery, 14 in health financing, and 11 in health resources purchasing. Of the 23 interviewed policy-makers and managers, 7, 9 and 7 were senior, middle and front-line managers, respectively. Three employees working at health ministry or medical universities were also interviewed.

**Table 1:** Demographic Characteristics of the Interviewees

	**Donors (n = 38)**		**Policy-Makers, Managers and Employees (n = 26)**		**Total (64)**	
	**No.**	**Percent**	**No.**	**Percent**	**No.**	**Percent**
Gender						
Female	12	31.6	8	30.8	20	31.2
Male	26	68.4	18	69.2	44	68.8
Marital status						
Single	6	15.7	3	11.6	9	14.1
Married	32	84.3	23	88.4	55	85.9
Age (year)						
25-35	6	15.8	3	11.6	9	14.1
36-45	4	10.5	10	38.5	14	21.9
46-55	10	26.3	2	7.6	12	18.7
Over 56	18	47.4	11	42.3	29	45.3
Education						
Under diploma	4	10.5	0	0.0	4	6.2
Diploma	9	23.7	0	0.0	9	14.1
Post diploma	5	13.2	0	0.0	5	7.8
Bachelor of science	7	18.4	5	19.2	12	18.7
Master of science	9	23.7	6	23.1	15	23.5
Doctor of medicine	1	2.7	9	34.6	10	15.6
PhD	3	7.8	6	23.1	9	14.1
Work experience						
1-10	12	31.6	3	11.6	15	23.5
11-20	9	23.7	11	42.2	20	31.2
21-30	6	15.8	9	34.6	15	23.5
Over 30	11	28.9	3	11.6	14	21.8

Brown and Clark’s six-step thematic analysis method including data familiarization, initial code identification, search for themes, review of themes, definition of themes and report preparation were used to analyze the qualitative data of this study.^13^ First, the content of the interviews was reviewed several times to gain a thorough understanding of the various aspects of the data. The initial codes were identified and extracted from the text of the interviews. Similar codes were placed in the sub-themes and finally in the main themes. The main and sub-themes were reviewed several times and, if necessary, combined, adjusted and separated to create a logical thematic map of the relationship between them. Then, the main themes and sub themes were defined. Finally, a report was produced using the identified themes, sub-themes, codes and interviewees’ quotations. The interviewed managers and donors were identified in this article with the letter M and D, respectively. Version 11 of the MAXQDA software was used for data analysis.

 Measures such as maximum diversity sampling, conducting pilot interviews, spending enough time to conduct interviews, examining the subject from different angles, gathering as much information and evidence as possible, constantly comparing the information obtained, submitting findings to a sample of interviewees and using their complementary comments and exchanging findings with peers were used to increase the credibility of the research. The research environment and stages were fully explained so that the reader could better judge the dependability and generalizability of the research findings. Obtaining the code of research ethics (IR.TUMS.SPH.REC.1396.3633) from Tehran University of Medical Sciences, voluntary participation, obtaining permission to record audio, keeping interviewees’ personal information confidential and neutrality of the researchers were ethical considerations followed in this study.

## Results

The challenges of donors’ participation in Iran’s health system were identified and grouped into 4 themes and 15 sub-themes (Table 2). Insufficient structures, poor communications, low trust, ineffective working processes, bureaucracy, insufficient senior managers’ support, weak legal support and weak monitoring were the most important challenges for donors’ participation in the health system. In addition, solutions to address these challenges were also provided. Effective donor participation in the health system requires the creation of an appropriate system including the right structures, processes, culture, and management.

**Table 2:** Challenges to Donors’ Participation in Iran’s Health System and Solutions to Address Them

**Themes**	**Challenges**	**Solutions**
Structural challenges	1. Inappropriate organizational structures 2. Poor intra and inter units communications	1. Establishment of a social deputy at the Ministry of Health 2. Establishment of a social deputy in medical universities 3. Establishment of healthcare charity support institutions 4. Establishment of non-governmental health charity organizations 5. Creating the national health charity networks 6. Effective communication between different units 7. Creation of facilitating committees 8. Signing a memorandum of understanding with related organizations
Process challenges	1. Ineffective working processes 2. Ineffective methods 3. Redundant bureaucracy 4. Weak monitoring and evaluation	1. Novel methods for collecting charitable donations 2. Benchmarking best practices 3. Establishment of a social deputy in medical universities 4. Training and empowering managers and employees 5. Developing an evaluation system
Cultural challenges	1. Weak communication between donors 2. Weak communication between health officials and donors 3. Low donors' trust in health officials 4. Donors’ immoral or illegal behaviors 5. Donors’ preference to spend their money in curative and rehabilitative services 6. Low participation of healthcare workers in philanthropic activities	1. Creating national charity networks 2. Publicity and public advertisement 3. Transparency of philanthropic activities 4. Appreciation of health donors 5. Training donors to fund health system priorities 6. Training health officials to treat donors well 7. Creating a database of health sector needs 8. Reinforcing the culture of philanthropic activities
Managerial challenges	1. Insufficient senior managers’ support and commitment 2. Insufficient health managers’ knowledge of donors’ capacities 3. Weak regulations, policies, and plans	1. Supportive laws, regulation and policies 2. Long- and short-term planning 3. Education and training of health managers and staffs

###  Structural Challenges

 Necessary organizational structures should be created to strengthen donors’ participation in the health system. In addition, the necessary cooperation should be established between various organizations inside and outside of the Ministry of Health.

####  Inappropriate Organizational Structures

 Interviewees noted that there are inadequate organizational structures in place for philanthropic giving. The lack of such organizational structures leads to several problems for donors. Interviewees suggested that establishing organizations such as social deputy at the Ministry of Health and medical universities, Health Donors Association, People’s Participation House and national networks of NGOs and charities, enhance donors’ participation in the health system and direct donors to the health system priorities.

Interviewees believed that there are thousands of individuals who are interested to donate into Iran’s health system. Therefore, there should be a society or an organization to mobilize and direct them to the health system priorities and address problems they may face during their philanthropic activities. Health Donors Association acts as the main NGO in Iran dealing with the health donations. Several sectors such as education and sport are competing to attract charity funds. Therefore, an organization should be created at the Ministry of Health to facilitate donors’ participation in financing the health system, generating resources and providing healthcare services. A donor said: *“We need appropriate organizations in medical universities and hospitals to handle the donors’ problems” *(D9). Another donor expressed: *“Hospital managers can create a structure, eg, a working group, for following up the donors’ problems. If a donor faces a problem, (s)he should be referred to that working group” *(D7).

The Social Deputy was established at the Ministry of Health in 2016. Consequently, a social deputy was established in medical universities and equipped with managers and staff to facilitate donors’ participation in the health system of each province of Iran. Such a structure was introduced at three levels, namely, Ministry of Health, medical universities and healthcare organizations. Accordingly, policies, procedures, plans and guidelines were developed. This structure made it possible for donors to have regular meetings with university authorities and direct their contributions to the needs and priorities of the health system. Each government hospital was asked to set up a hospital support charity. A senior manager in social deputy of the health ministry said: *“Each public medical center should have a supportive charity. Hospital chief executive officer should attend the *[charity’s]* board meetings, hospital needs should be discussed there and donations should be directed towards hospital priorities”* (M9). About 84% of public hospitals in the country created their own supportive charities.

Furthermore, building structures such as People’s Participation House, Neighbourhood Health Centre and health-related non-governmental organizations were useful in enhancing donors’ participation in the health system and directing their philanthropic actions towards the health system priorities. A senior manager at the Ministry of Health said: “*Donors can attend People’s Participation Houses. The purpose of creating such structures is to empower people, educate them and promote their health. Donors’ awareness and knowledge about health will be enhanced there” *(M1). A manager in Iranshahr Medical University said: “*Doctors and volunteers attend health NGOs and visit patients for free there” *(M10).

Creating national networks of NGOs and charities is another effective solution to enhance donors’ participation. When a donor sees his/her donation as part of a national charity network, (s)he is motivated to donate again and more. The dean of Larestan medical school believes: *“Creation of national networks of health donors is a good idea for enhancing donors’ participation. They learn about each other’s [philanthropic] activities. They may become interested in helping in other areas or other cities” *(M15). A member of the national network of cancer treatment donors also said: *“Creating a national network of health donors was an excellent solution. Donors gather from different parts of the country. They discuss health issues and become more aware” *(D14).

####  Poor Intra- and Inter-units Communications

Financing and delivery of health services are complex and complicated and requires coordination among several bodies inside and outside of the health system. Donors are not aware of such a complex system and as a result face some challenges. A hospital manager said: *“Donors face some problems in the construction of a charity medical center, particularly because of the weak communication between different units involved in this process. Effective communication between these units may reduce the donors’ confusion” *(M16).A manager at Ministry of Health said: *“Poor communication between a non-governmental charity organization and the government is a big challenge. There is no proper communication between them” *(M7). A donor in Kurdistan province stated: *“I was disappointed when I built a clinic in collaboration with the university. The university did not cooperate well” *(D13). Weak communications in health and medical organizations lead to weak donors’ participation in the health system. Creation of facilitating committees in medical universities and healthcare organizations can improve such intra- and inter-unit communications.

Some interviewees suggested that developing relationship with other related national and international organizations facilitate further donors’ participation in the health system: *“Voluntary and charity activities were not formed in all groups of the society. There is a need for more cooperation with other organizations such as the Youth Organization and the Red Crescent Volunteers Organization” *(M7).* “Some of the problems of universities in using endowments were solved through the signing of a memorandum of understanding between the Ministry of Health and the Endowment Organization in 2014. The memorandum of understanding with the Ministry of Foreign Affairs was also useful in attracting charitable donations from Iranians living abroad” *(M9).

###  Process Challenges

 Ineffective work processes, ineffective methods, bureaucracy and weak monitoring and evaluation are examples of procedural barriers to donors’ participation in the health system.

####  Ineffective Working Processes

Ineffective working processes cause confusion and frustration for health donors. A donor in the city of Hamedan said: *“One of our problems at the charity hospital is the working processes of related organizations, especially the health insurance organization. Their procedural problems have a negative effect on the quality of our hospital services” *(D5). A manager at Ministry of Health said: *“Donors face many problems in building medical centers. Most problems are due to the lack of a proper work process. We need to define the right processes” *(M13).

Interviewees suggested that establishing the social deputy department at the Ministry of Health has been a good way to evaluate existing processes, find the bottle necks and improve them: *“Since the establishment of the Social Affairs Department of the Ministry of Health in 2016, we have been working on work processes related to the participation of donors in the health system. We have organized and improved them” *(M21).

####  Ineffective Methods

As the science and technology advance, the methods of collecting charitable donations in the health system must also change. A donor in Tehran said: *“There are a lot of donors. Their participation should be managed systematically, not in a traditional way” (*D10). Websites and virtual social networks can be used to raise money for charity. A health manager believed that:* “Some donors do not like traditional financing methods such as charity cash boxes or fundraising. They prefer new methods such as media and virtual networks” *(M25).A manager at Health Ministry also suggested: *“An electronic system should be established at the Ministry of Health to show donors the needs and priorities of the health system” *(M9). Such information structures direct donors’ financial contribution to the priorities of the health system. A health manager in the province of Sistan expressed that: *“There are few donors in poverty-stricken areas. When the needs of the people of this region are introduced through virtual media, donors from other parts of the country become interested in helping out” *(M2).Another senior manager at the Health Ministry believed: *“A lot of issues can be introduced through national media, and a lot of help can be attracted from the public” *(M7).

Some interviewees found benchmarking best practices useful for attracting health donors. A manager at the Ministry of Health said: *“We must use the successful experiences of other countries to strengthen the participation of health donors. We should adopt others’ methods and adapt them to our context and culture” *(M7). Managers cited examples of the benchmarking other cities’ and countries’ methods: “*Accommodations were built by donors for families with cancer patients to stay near the hospital in Tehran. The idea came from McDonald House Charities” *(M18).* “Health donors raised about 12 million Tomans [US$2850] through a campaign for a hospital in Isfahan. The idea of raising money through the campaign was new. The campaign was welcomed by the people” *(M17).

####  Redundant Bureaucracy

Health donors cited many examples of existing bureaucracies that hindered their participation in the health system: *“I encountered many obstacles to buy an electro-shock device for a hospital in Tehran. Hospital managers did nothing to speed up the process” *(D10). “*I decided to build a hospital in Tehran. However, due to the poor cooperation of the medical university, it has not been equipped yet” *(D9). “*I had a lot of problems getting a license to set up a medical center. Approval of various departments must be obtained which is time consuming” *(D19).* “The university gave me an unsuitable plot of land to build a clinic. I encountered many problems in installing its mechanical and electronic facilities, and the university did not cooperate much with me” *(D13).* “I bought a medical device for a hospital months ago. I have not received it from customs yet. We face many problems when buying specialized hospital equipment” *(D4).

A senior manager in the Health Ministry believed that: *“Donors with good intentions want to help the health sector. We need to facilitate and expedite their work” *(M8). Leaving donors alone to handle these problems may disappoint them and direct their charities to other areas.A donor in the city of Tehran said: *“Donors will be disappointed if they see many obstacles in their charitable activities in the health sector” *(D6).A senior manager at the Health Ministry also said: *“Donors face a number of problems while contributing to the health sector that may frustrate them” *(M3).

Building and equipping a hospital and clinic is costly and time consuming and requires obtaining a license from various authorities. An organization at the Ministry of Health and the medical university should help donors in this regard. One of the health donors suggested: “*Establishing a charity office at the university and hospital that liaises with donors will help improve processes and reduce administrative bureaucracies” *(D7).Furthermore, training and empowering the managers and staff of the social deputy of universities and medical centers while accelerating the charitable affairs will increase donors’ satisfaction: “*Sometimes bureaucracy is inevitable. But employees must explain the process to donors, and advise them on the required documents. Proper counseling and interaction helps donors. Our managers and employees need to be trained in this area”* (M16).

####  Weak Monitoring and Evaluation 

Interviewees believed that insufficient control led to poor awareness of the performance of donors’ participation in the health system, and as a result, a failure to take timely corrective measures to strengthen their participation. An interviewee at Ministry of Health said: *“There has not been an effective system for evaluation of the donors’ participation. Donors’ resources can be used purposefully using a monitoring and evaluation system” *(M9).

Donors’ participation in the health sector should be monitored and evaluated at macro and micro levels. Evaluation leads to the prevention of waste of resources and better management of charitable resources: “*We need a proper monitoring and evaluation system for the activities of health charities. Indicators and evaluation checklists should be properly and comprehensively designed and used”* (M21).* “The dashboard system provides regular feedback to universities on charitable activities in their areas. Accordingly, they can take the necessary steps to increase donor participation”* (M18).

###  Cultural Challenges 

 In theory, good structures and processes will result in good outputs and outcomes. However, in practice, there are several underlying contextual factors that affect the results. Interviewees identified a number of contextual and cultural challenges that prevented donors from participating in the health system such as poor communication, low trust, donors’ immoral behaviors, their preference to contribute more to medical services, and healthcare workers’ low participation in philanthropic activities.

####  Weak Communication Between Donors 

Poor communication between donors leads to rework and parallel work. A senior manager in the Larestan medical school said: *“A donor built a clinic in our area. Sometime later, another donor requested the construction of a similar clinic here” *(M15). Insufficient knowledge of donors about the health needs of the region causes these events:* “Donors do not know that they can participate in areas other than building hospitals and clinics too. They can participate in screening, prevention, public health, and vaccination services. They should be informed” *(M1).* “Donors have little incentive to participate in prevention, education and research due to low awareness of the real needs of the health system”* (M3).There is a weak link between donors and charity organizations. Creating national charity networks will strengthen the connections between them.

####  Weak Communication Between Health Officials and Donors

Poor communication between health officials and donors also poses some challenges. A donor in Kurdistan said: “*I decided to build a specialized diabetes clinic for the university. I had to build the clinic and the university had to provide land and equipment. University cooperation was weak. When I built the clinic, the staff and equipment were not ready yet. Sometimes I get frustrated” *(D13). A donor in the province of Fars also said: *“The tax officer did not treat me well when I asked for a tax deduction for a charity project” *(D11).

The interviewees believed that healthcare providers should treat health donors well. They should be trained in this regard: *“The managers’ perspective is to get only financial support from the donors. They [managers] should be trained to treat them well too” *(M26).“*We should educate and empower our managers to organize donors’ participation in the health sector” *(M22). *“Donors are benevolent people. They should be treated accordingly. They should be given proper advice. I saw that the inappropriate behavior of the healthcare officials upset them” *(M8).

####  Low Donors’ Trust in Health Officials

Poor communication between health authorities and donors leads to mistrust. A medical school dean believed that:* “Donors have little trust in government agencies in managing their charitable contributions. They want to build hospitals and medical centers for the government, so they expect their financial resources to be used properly” *(M10). A donor in the city of Tehran said: *“Some donors have little trust in the government. Therefore, they prefer to give their money to trusted people to spend in a good way” *(D33). One of the senior managers of Zabol medical university also said:* “Donors’ distrust in the government is a great challenge. I tried to gain their trust and I was able to increase their participation in the health sector in this region” *(M2).

Advertisement, transparency and appreciation of health donors are good ways to strengthen their contribution to the health sector: “*Publicity and public advertisement are useful to gain the trust of health donors” *(M24). *“Donors have recently had widespread contributions to the medical centers and medical universities. Managers should advertise donor’s efforts to the public” *(M6). *“Making a television documentary and broadcasting it in the national media strengthen the culture of donations. We have chosen 40 health donors and are making a TV documentary about their lives and their philanthropic activities in health sector” *(M9).

####  Donors’ Immoral and Illegal Behaviors

Some donors may contribute to the health system for illegal purposes such as money laundering, tax evasion, and illegal requests. A medical school manager said: *“Health donors sometimes make illegal requests. They may build a medical center and ask the university to employ their relatives there” *(M10). A donor also confirmed this: “*After the construction of a medical center, a donor asked the university authorities to employ one of his relatives there” *(D17).

Interviewees believed that proper rules and regulations and continuous monitoring and evaluation will reduce these illegal behaviors. Some interviewees believe that: “*Immoral and illegal behaviors will be reduced through establishing right and transparent rules and proper monitoring and evaluation. We must have more control while facilitating the process of donors’ participation in the health sector*” (M21). “*Transparency, accountability, and trust-building reduce donors’ illegal and immoral behaviors. We need good supervision, so that the donors do not deviate from the right path*” (M3).

####  Donors’ Preference to Spend Their Money in Curative and RehabilitativeServices

Some interviewees argued that donors’ resources are not fairly distributed in the health sector. They prefer to contribute more to curative and rehabilitative services: *“Most donors are only interested in building medical centers. They *[donors]* are less interested in contributing to the prevention and education areas. Also, most donors want to help in specific geographical areas. They [donors] do not contribute to the health needs of the poor areas. They are more interested in building specialty hospitals in metropolitans” *(M3). “*Donors rarely contribute to research. Health system also requires research and development. The country also needs the help of donors in the field of medical research” *(M6). *“Donors should also contribute to the field of medical education. They [donors] can contribute to financing the education of poor medical and paramedical students. Their participation should not be limited to building medical centers” *(M15).

A manager at the Health Ministry believed that: *“The Health Ministry itself has acted weakly in planning and managing donors’ contribution, and that is why they [donors] only like to build hospitals and clinics” *(M14). Creating a database of health sector needs directs donors’ resources to the needs of the health sector: *“Creating a database of the needs of health, education and research will lead to better guidance of donors to the real needs of the health sector. They [donors] will be familiar with the needs of all health sectors and will not participate in just one specific area” *(M12).

####  Low Participation of Healthcare Workers in Philanthropic Activities

Health workers, such as doctors and nurses should be at the forefront of humanitarian aid. Some health donors believed that: *“a culture of donation should be promoted in the health system too. For example, if 500 million Tomans *[US$ 118750]* is to be collected, doctors should also help financially”* (D23).* “Health managers and doctors should also donate to charity. They should not just wait for the help of others” *(D3). A volunteer doctor also said: “*After finishing my studies, I went to a deprived area. In addition to visiting patients there, I perform other voluntary activities such as providing healthy nutrition. Other doctors can also help improve the health of people in disadvantaged areas” *(D20).Involvement of health workers in philanthropic activities motivates other people to participate more in these activities.

###  Managerial Challenges

 Many of the structural, process, and cultural challenges outlined above are due to managerial and governance issues such as insufficient managers’ knowledge and support and poor legal support.

####  Insufficient Senior Managers’ Support and Commitment

Senior health managers should understand the importance of donors’ contribution and support them. A health donor in Tehran said: “*Poor management and lack of senior managers’ support waste donors’ resources*. *Managers who work in the health charity offices must be experienced and knowledgeable*” (D12). A manager at Ministry of Health said: *“10 years ago, the university health deputy did not support donors and charities. Many charitable projects were stopped at that time. However, his successor supported donors’ participation, and we have had a lot of success in this area”* (M9). Another manager at Health Ministry also said: *“Donors cannot implement major projects alone. They [donors] need our [managers’] support” *(M21).

####  Insufficient Health Managers’ Knowledge of Donors’ Capacities

Health managers should be aware of the donors’ capacities and resources. A senior manager at Ministry of Health said: *“Most managers’ perspective is to receive financial resources from the donors. The donors’ capacities are much wider than that. Managers’ attitudes must change” *(M1). A donor in the area of cancer treatment said: *“Government managers see donors as business enterprises, from whom they can obtain financial resources” *(D14). Asenior manager at health ministry said:* “Those managers and staff working at health charity offices in universities should be trained and empowered to enhance donors’ participation in the health system” *(M5).

####  Weak Regulations, Policies and Plans

Donors’ participation in the health system requires legal support. Although there are laws, regulations and policies to support donors, some of them prevent donors from contributing to the health sector. For example, new tariff for public, private and charity hospitals’ services were announced by the Ministry of Health. Some donors have argued that the low tariff for charity hospital services does not motivate them to provide health services: *“Hospital costs are high. The hospital cannot be run with this low tariff. We [donors] need the support of the law to increase hospital tariffs” *(D7).* “Hospital tariffs must be set fairly so that we can provide quality health services” *(D6).

According to Article 172 of the Law on Direct Taxes, all funds spent by individuals on the construction, repair and equipment of schools, universities and hospitals in accordance with the rules of the Ministries of Education, Science and Health will be exempt from tax. Donors see this law as an incentive to get more involved in humanitarian work. They also expected to be exempt from 9% value added tax (VAT): “*I bought a medical device worth 88 million Tomans [US$ 20 900] for a hospital in Tehran. I had to pay the tax for this device. The government can support donors by exempting them from VAT*” (D7). VAT exemption is a good incentive for donors to purchase more equipment for healthcare organizations.

Several policies were developed at the Health Ministry to motivate donors to contribute more to the health system. For example, all medical universities and healthcare organizations were asked to affix license plates on donated properties and equipment in 2017, to honor their philanthropic actions and make it transparent. Furthermore, a strategic plan is needed to strengthen philanthropic actions in the Iranian health system. A manager in a university said: “*We need a strategic and operational plan to strengthen donors’ philanthropic activities*” (M9). A donor had the same remark: *“Health Ministry should have comprehensive plans. Donors need to be educated and guided by expert organizations. There should be a framework for their participation” *(D4). Some interviewees believe that donors’ contribution should not only be limited to financing, resource generation and healthcare delivery. They should be involved in policy-making and planning too.

###  A Conceptual Model for Strengthening Donors’ Participation in Healthcare

 The health system is very complex and includes institutions, groups and individuals involved in policy making, financing, generating resources, and providing health services to maintain, restore and promote people’s health. Policy-makers, payers, patients and healthcare providers are the key components of the health system. The health system is surrounded by an external environment that includes political, economic, social, cultural, technological, and moral factors that affect it. About 80% of people’s health is, due to those factors outside the health system. Therefore, a good interaction must be established between the health system and the surrounding environment.

 Donors can help finance the health system, generate resources, provide health services, and even manage the health system. Health policy-makers and managers must take a systemic approach to strengthening donors’ participation in the health system. In systems theory, inputs and processes must be improved and enhanced to achieve good outputs and outcomes. A structure should be established at the Ministry of Health to mobilize and coordinate health donors. Such a structure should be formed in the medical universities and the healthcare organizations. Health system priorities should be set and communicated to donors. Laws, regulations and supportive policies should be developed to facilitate the participation of donors in the health system.

Strengthening the participation of donors in the health system requires a long-term strategic plan and annual operational plans. The goals of donors’ participation and strategies to achieve them should be clearly defined. The necessary resources must be provided for implementing those plans and the coordination between key stakeholders should be improved. Policy-makers, managers and health donors need to be trained and empowered to play their role better. The necessary changes to promote donors’ participation in healthcare must be planned and led. The performance of health donors should be evaluated continuously, and corrective actions should be taken if necessary. In this case, we will see more and better participation of donors in financing, producing resources and providing health services (Figure).

**Figure:**
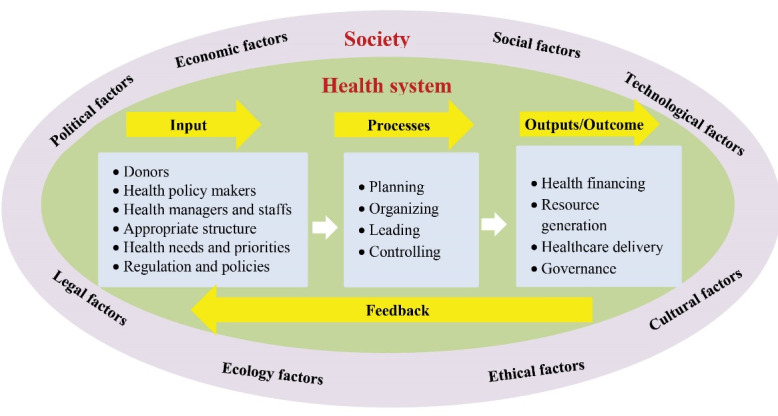
A Model for Strengthening Donors’ Participation in the Health System.

## Discussion

 The purpose of this study was to identify barriers to donors’ participation in Iran’s health system and to provide solutions. Donors face structural, process, contextual, and managerial challenges when financing, generating resources, and providing health services. Insufficient structures, poor communications, low trust, ineffective work processes, bureaucracy, insufficient senior managers’ knowledge and support, weak legal support and weak monitoring were the most important challenges for donors’ participation in the health system.

Donors are directly involved in providing health services and indirectly in providing support services such as building hospitals and health centers and purchasing medical equipment in the Iranian health system.^6^ Their participation is mainly individual and unorganized. Many donors are interested in contributing to the Iranian health system. Their participation must be organized and coordinated to achieve better results. Unfortunately, the necessary structures for mobilizing donors are not sufficiently established in the health system. The formation of the Ministry of Health’s social deputy in 2016 has been the first effective step in planning, organizing, leading and directing health donors. As a result, an administration in medical universities took over the management of health-related charities in the country’s provinces. The social deputy at the Ministry of Health was dissolved in 2020 and reduced to the level of a general administration in health charities. This change has had negative effects on donors’ participation in the health system.

 An appropriate structure with sufficient resources should be established at country, province and city levels to take responsibility for planning, organizing, leading and controlling activities related to health donors. Financing, generating resources and providing health services require coordination and cooperation of different sectors inside and outside the health system. First, associations should be created so that donors can have more effective group activities by joining them. Second, an office should be established at the Ministry of Health and medical universities to facilitate donors’ participation in the health sector.

 Donors help the government in providing public services by investing in the public sector. Therefore, a structure must be created in the government to strengthen the participation of donors in public services. This structure will provide the necessary support for the Health Donors Office of the Health Ministry. As a result, intra-departmental communication is facilitated and donors’ participation in major healthcare projects is strengthened and accelerated.

Ineffective working processes, inappropriate methods, bureaucracy and poor evaluation were process challenges of donor participation in the Iranian health system. Modern and innovative methods should be used to attract donors’ donations in the health system. Bureaucracy is a major obstacle to the participation of health donors and sometimes discourages them. Improving work processes and eliminating unnecessary bureaucracy increases donors’ participation in the health sector.^14^ Donor participation processes need to be identified, standardized and improved so that donors can carry out health projects easily and quickly. Supportive laws and regulations should be developed to encourage the participation of health donors. Furthermore, the level of donors’ participation should be evaluated regularly and compared to the set goals so that corrective measures can be taken if necessary.

 Poor communication between donors themselves, poor communication between donors and health authorities, low donors’ trust in health officials, illegal behavior of donors, low donors’ willingness to participate in non-medical affairs and low participation of health workers in philanthropic activities are cultural barriers to donors’ participation in the health system.

Philanthropic behavior is an activity performed by a person with the aim of helping people in need.^15^ Healthcare donors follow a purposeful and planned behavior for contributing to the health system. Donors’ participation in the health system is a function of their goals, intentions, attitudes, and their control of perceived behavior. The donors’ attitude is shaped by their characters, personality, background knowledge, experience, past behavior, and norms.^16^ The nature of work is a motivating factor that leads to people’s satisfaction.^17^ The nature of altruistic health services plays an important role in attracting donors to participate in the health system. Hence, healthcare managers should build and cultivate good relationships with donors. Keeping in touch with donors, informing them about the needs and priorities of the health sector, giving them feedback on how to spend their financial aid, and recognizing them will enhance their motivation to participate more in the health sector.

 Finally, managerial barriers such as insufficient legal support, limited health managers’ support and knowledge of health donors’ capacities create many problems for donors’ participation in the health system. Health donors face many challenges from the formation of a health charity to the financing, generating health resources and provision of health services. According to the Article 65 of the Sixth Five-Year Development Plan, the Government of Iran is obliged to increase the share of donors’ financial contribution through proper legislation and planning. Further supportive laws and regulations should be developed to encourage donors to participate in the health sector.

 Unequal distribution of health donors in the country and economic difficulties lead to instability of donors’ contributions to the health system. Today we face many political, economic, social, cultural, environmental and epidemiological changes that sometimes pose threats to the society. Traditional development models are not very effective in eliminating poverty and reducing inequity. Therefore, politicians must pay attention to sustainable development. Sustainable development requires real people participation in economic, social, cultural and welfare programs. The informed and voluntary participation of people in the planning, implementation and evaluation of health programs, enhances their commitment to health programs’ effective implementation. As a result, it will be possible to achieve equity in universal health coverage, especially for the deprived regions of society. Therefore, people in general and health donors in particular need to be educated about the health system, and its functions, goals and strategies.

Healthcare organizations are facing many internal and external changes. Strategic management and planning helps health managers better maintain their internal cohesion and adapt to the external environment. ^18^ Health policy makers and managers should utilize donors’ resources by anticipating the healthcare needs of the country and formulating a strategic plan. Managers should inform donors of the country’s health needs and use their resources to provide effective and efficient health services. Top managers’ stability encourages long-term planning and commitment to pursuing long-term objectives.^19^

However, some people may contribute to the health sector with immoral and illegal motives such as money laundering, tax evasion and unnecessary requests. Philanthropy should not affect the care provided to patients. Clear distinctions are needed between health donors and health providers.^20^ Health managers should prevent these problems through the establishment of rules and regulations and continuous monitoring and evaluation.

 This study identified the challenges of donors’ participation in the Iranian health system using inductive approach and pluralistic evaluation. In addition, solutions were proposed to address these challenges. Future studies can measure these challenges using a questionnaire through a deductive approach. Health policy-makers and managers in other countries can use the proposed solutions to strengthen the participation of donors in their health system.

## Acknowledgements

 We appreciate the support of managers and staff at the Social Deputy of Iran’s Health Ministry during the data collection. We are thankful to all the health managers and donors who participated in interviews and shared their knowledge, experience and insight. We are grateful to the IJHPM editorial team and reviewers for their constructive comments.

## Ethical issues

 This study approved by Tehran University of Medical Sciences (ethics No. IR.TUMS.SPH.REC.1396.3633).

## Competing interests

 FE used to work at Social Deputy of Iran’s Health Ministry. However, all authors followed all ethical consideration and this working relationship did not affect the result of the study. All authors were neutral at collecting, analyzing and interpreting the data. Authors declare that they have no competing interests.

## Authors’ contributions

 All authors conceptualized, drafted and edited the manuscript. FE collected the Data. AMM and FE analyzed the data. All authors approved the final version of the manuscript.

## References

[1] Iran Statistical Center. https://www.amar.org.ir/english. Accessed November 17, 2020.

[2] The World Bank. https://data.worldbank.org. Accessed January 6, 2020.

[3] Mosadeghrad AM. Health transformation plan in Iran. In: Braithwaite J, James W, Ludlow K, eds. Health Systems Improvement Across the Globe: Success Stories from 70 Countries. Taylor & Francis. 2017:309-316.

[4] Mosadeghrad AM, Ferlie E. Total quality management in healthcare. In: Management Innovations for Healthcare Organizations: Adopt, Abandon or Adapt? York: Routledge. 2016:378-396.

[5] Eikenberry AM (2005). Fundraising or promoting philanthropy? a qualitative study of the Massachusetts Catalogue for Philanthropy. Int J Nonprofit Volunt Sect Mark.

[6] Mosadeghrad AM, Tajvar M, Ehteshami F. Donors’ participation in healthcare delivery in Iran. Payesh (Health Monitor) 2019;18(5):438-453. [Persian].

[7] World Health Organization (WHO). Global Spending on Health: Weathering the Storm. Geneva: WHO; 2020.

[8] Charities Aid Foundation. World Giving Index 2018: A Global View of Giving Trends. https://www.cafonline.org/docs/default-source/about-us-publications/caf_wgi2018_report_webnopw_2379a_261018.pdf. Accessed March 28, 2021.

[9] Ayazi MH, Jamali M, Javadi MH, Hoseini Nejad J, Rafiefar S, Zamani Garmsiri S, et al. Deputy for Social Affair at a Glance, Ministry of Health and Medical Education. Tehran: Barta Publication; 2018. p. 18. [Persian].

[10] Mosadeghrad AM, Tajvar M, Ehteshami F. Donors’ participation in financing health system of Iran. Hakim Health Syst Res 2019;22(1):26-42. [Persian].

[11] Mosadeghrad AM, Janbabaei G, Kalantari B, Darrudi A, Dehnavi H. Equity in distribution of hospital beds in Iran. Sci J Kurdistan Univ Med Sci 2020;24(6):12-36. [Persian].

[12] Husserl E. Phenomenology and the Foundations of the Sciences. Netherlands: Springer Science & Business Media, 2001:21-43.

[13] Braun V, Clarke V (2006). Using thematic analysis in psychology. Qual Res Psychol.

[14] Aghababa S, Nasiripour AA, Maleki M, Gohari M (2017). Donor retention in health care in Iran: a factor analysis. Med J Islam Repub Iran.

[15] Alias SN, Ismail M (2015). Antecedents of philanthropic behavior of health care volunteers. Eur J Train Dev.

[16] Mosadeghrad AM, Ehteshami F. Explaining and Predicting Donors’ Participation Behavior in Iranian Health System. Hakim Health Syst Res 2019;22(4):284-297. [Persian].

[17] Mosadeghrad AM, De Moraes A (2009). Factors affecting employees’ job satisfaction in public hospitals: implications for recruitment and retention. J Gen Manag.

[18] Esfahani P, Mosadeghrad AM, Akbarisari A (2018). The success of strategic planning in health care organizations of Iran. Int J Health Care Qual Assur.

[19] Mosadeghrad AM, Ferdosi M, Afshar H, Hosseini-Nejhad SM (2013). The impact of top management turnover on quality management implementation. Med Arch.

[20] Kalina P (2020). Ethical Philanthropy in the era of Patient-Centric, Value-Based Health Care. Arch Bus Res.

